# The promise of adult neurogenesis for treating and preventing chronic neurodegenerative diseases

**DOI:** 10.31491/apt.2024.09.147

**Published:** 2024-12-26

**Authors:** Gerald Yu Liao, Warren Ladiges

**Affiliations:** a Department of Comparative Medicine, School of Medicine, University of Washington, Seattle, WA 98195, USA.

**Keywords:** Adult neurogenesis, neurodegenerative diseases, Acheta domesticus, immunohistochemistry, RNA sequencing

## Abstract

Chronic neurodegenerative diseases pose a significant public health challenge due to their profound impact on individual autonomy and identity. Adult neurogenesis (ANG), the ongoing generation of neurons in specific brain regions, offers promising therapeutic avenues for these diseases. Despite controversies surrounding the existence and functional relevance of ANG in humans, emerging evidence suggests individual variability in ANG induction may hold the key to understanding and addressing these conditions. We advocate for a shift in research focus towards unraveling the genetic mechanisms governing ANG and understanding its functional significance in cognition. Novel models are needed to move this concept forward. The heterogeneous domestic house cricket (*Acheta domesticus*) has practical advantages and potential for rapid insights into neurogenesis. This model, coupled with advanced methodologies such as immunohistochemistry and RNA sequencing, can provide a detailed understanding of ANG and its therapeutic potential. Ultimately, embracing innovative models and holistic approaches to ANG research will help unlock new strategies for treating and preventing chronic neurodegenerative diseases.

Chronic neurodegenerative diseases are a major public health problem due to their profound impact on individual autonomy and identity. These diseases cause a gradual loss of self-recognition and essential functions, ultimately leading to death. The burden extends beyond affected individuals to their families and society, resulting in substantial medical costs, caregiver demands, and emotional tolls. Effective solutions would alleviate personal suffering and reduce societal and economic impacts, allowing for a more natural and dignified aging process free from debilitating symptoms.

The phenomenon of adult neurogenesis (ANG) has captivated the scientific community with its implications for brain plasticity and potential therapeutic avenues. However, amidst the excitement lie many controversies and unanswered questions surrounding its existence and functional significance in humans.

Human ANG, the ongoing generation of neurons in specific brain regions beyond neonatal development, primarily occurs within the sub-granular zone (SGZ) of the dentate gyrus (DG) in the hippocampus and the subventricular zone (SVZ) lining the lateral ventricles [1]. Despite its apparent simplicity, the topic remains shrouded in debate, with conflicting findings stemming from decades of research.

Initial discoveries relied on methodologies such as 5-bromo-2’-deoxyuridine (BrdU) labeling and carbon-14 dating, but their limitations in specificity and interpretation have fueled skepticism. Immunohistology staining techniques, while promising, face challenges in standardization and reliability due to factors such as postmortem delay and subject characteristics. Even with advancements like single nuclear RNA-sequencing, contradictory results persist, hinting at the underlying complexity of ANG regulation.

However, amidst the noise of debate, a compelling narrative emerges: individual variability in ANG induction may hold the key to understanding neurodegenerative diseases. Preliminary evidence suggests altered rates of ANG in conditions such as Alzheimer’s Disease (AD) and epilepsy, pointing towards underlying heterogeneity in genetic predispositions. Instead of fixating on the existence of ANG, future research should pivot towards unraveling the genetic mechanisms governing its induction, differentiating individuals who exhibit evidence of ANG from those who do not. Moreover, beyond its mere existence, the functional relevance of ANG in human cognition remains a pivotal question. Does an increase in mature neurons necessarily translate to improved cognitive function? Insights into developmental disorders such as Rett syndrome highlight the importance of synaptic connectivity over neuronal numbers, urging for a holistic approach to studying ANG’s impact on brain function.

Better models are needed. For example, the domestic house cricket (*Acheta domesticus*) presents a compelling avenue for research due to several key factors. In house crickets, ANG is observed within the mushroom bodies (MBs), which serve as the primary multimodal integrative center of information [2]. Neurogenesis in the MBs involves interneurons known as Kenyon cells, originating from neuroblasts clustered at the cell cortex apex [2], analogous to neurogenic stem cells (NSCs) in the mammalian hippocampus [1]. This process is intricately influenced by olfactory and visual inputs, as well as connections to efferent fibers projecting to various brain structures [2]. Perturbations in sensory structures can disrupt neuroblastic proliferation, highlighting the model’s sensitivity to environmental cues. Studies utilizing this insect model have probed the behavioral consequences of inhibiting ANG through targeted lesions, revealing insights into cognitive decline. Notably, behavioral assays such as the “escape paradigm” have elucidated the impact of ANG on learning and memory, with crickets lacking ANG exhibiting delayed learning and impaired memory retention [2]. Such findings underscore the cricket model’s potential for unraveling the complexities of neurogenesis and its implications for cognitive function.

Beyond its scientific merit, the house cricket model boasts practical advantages: its heterogeneous simplicity, rudimentary but well-defined organ systems, shared diet with humans, and ease of rearing in large quantities make it a cost-effective and accessible model for studying ANG [3]. Furthermore, with its short life cycle and distinct developmental stages, the house cricket promises rapid insights into aging and the intricate mechanisms of neurogenesis.

By employing immunohistochemistry with specific antibodies, we can confirm the presence of various cell types involved in ANG, such as neural progenitors and mature neurons. Additionally, harnessing the ability to culture Kenyon cells would allow us to trace the development and integration of new neurons as well as map the influence of *in vitro* drug testing. RNA sequencing, both single-cell and bulk, can provide a detailed transcriptomic landscape, revealing the molecular mechanisms underlying ANG. This model also holds promise for drug testing aimed at treating mild cognitive impairment and Alzheimer’s disease (AD), using adeno-associated viral vectors to introduce AD-related genes and assess the impact on neurogenesis and neuronal health. Behavioral tests, including cognitive assessments and locomotor activity measurements, have been validated and can evaluate the functional effects of neurogenic interventions.

In conclusion, the study of ANG transcends mere scientific curiosity, as it holds the promise of unlocking new therapeutic strategies for neurodegenerative diseases. By embracing innovative methodologies and unconventional models such as the house cricket, there is potential for navigating the complexities of ANG towards a deeper understanding of brain plasticity and function, providing new insights into ANG and potential therapeutic strategies for neurodegenerative diseases.

## Data Availability

Not applicable.
